# Identification of Interleukin-1-Beta Inhibitors in Gouty Arthritis Using an Integrated Approach Based on Network Pharmacology, Molecular Docking, and Cell Experiments

**DOI:** 10.1155/2022/2322417

**Published:** 2022-09-19

**Authors:** Liying Zeng, Zekun Lin, Pan Kang, Meng Zhang, Hongyu Tang, Miao Li, Kun Xu, Yamei Liu, Ziyun Jiang, Shaochuan Huo

**Affiliations:** ^1^Guangzhou University of Chinese Medicine, Guangzhou 510405, China; ^2^College of Basic Medicine, Guangzhou University of Chinese Medicine, Guangzhou 510006, China; ^3^Department of Joint Orthopaedic, The First Affiliated Hospital of Guangzhou University of Chinese Medicine, Guangzhou 510405, China; ^4^Lingnan Medical Research Center of Guangzhou University of Chinese Medicine, Guangzhou 510405, China; ^5^Department of Orthopedics, Henan Provincial People's Hospital, People's Hospital of Zhengzhou University, People's Hospital of Henan University, Zhengzhou 450003, China; ^6^Shi's Center of Orthopedics and Traumatology, Shuguang Hospital, Affiliated to Shanghai University of Traditional Chinese Medicine, Institute of Traumatology & Orthopedics, Shanghai Academy of Traditional Chinese Medicine, Shanghai, China; ^7^Shenzhen Hospital (Futian) of Guangzhou University of Chinese Medicine, Shenzhen 518048, China

## Abstract

**Background:**

This study aimed to investigate the molecular mechanism of Tongfengding capsule (TFDC) in treating immune-inflammatory diseases of gouty arthritis (GA) and interleukin-1-beta (IL-1*β*) inhibitors by using network pharmacology, molecular docking, and cell experiments.

**Methods:**

In this study, the compounds of TFDC and the potential inflammatory targets of GA were obtained from Traditional Chinese Medicine Systems Pharmacology Database and Analysis Platform (TCMSP), Online Mendelian Inheritance in Man (OMIM), and GeneCards databases. The TFDC-GA-potential targets interaction network was accomplished by the STRING database. The TFDC-active compound-potential target-GA network was constructed using Cytoscape software. Gene Ontology (GO) and Kyoto Encyclopedia of Genes and Genomes (KEGG) pathway enrichment analyses were used to further explore the GA mechanism and therapeutic effects of TFDC. Quantitative real-time PCR (qPCR) was used to verify whether the TFDC inhibited IL-1*β* in GA. Molecular docking technology was used to analyze the optimal effective compounds from the TFDC for docking with IL-1*β*.

**Result:**

133 active compounds and 242 targets were screened from the TFDC, and 25 of the targets intersected with GA inflammatory targets, which were considered as potential therapeutic targets. Network pharmacological analysis showed that the TFDC active compounds such as quercetin, stigmasterol, betavulgarin, rutaecarpine, naringenin, dihydrochelerythrine, and dihydrosanguinarine had better correlation with GA inflammatory targets such as PTGS2, PTGS1, NOS2, SLC6A3, HTR3A, PPARG, MAPK14, RELA, MMP9, and MMP2. The immune-inflammatory signaling pathways of the active compounds for treating GA are IL-17 signaling pathway, TNF signaling pathway, NOD-like receptor signaling pathway, NF-kappa B signaling pathway, Toll-like receptor signaling pathway, HIF-1 signaling pathway, etc. The TFDC reduced IL-1*β* mRNA expression in GA by qPCR. Molecular docking results suggested that rutaecarpine was the most appropriate natural IL-1*β* inhibitor.

**Conclusion:**

Our findings provide an essential role and bases for further immune-inflammatory studies on the molecular mechanisms of TFDC and IL-1*β* inhibitors development in GA.

## 1. Introduction

Gouty arthritis (GA), one of the most serious and common arthritis usually starting suddenly at night, is a lesion and inflammatory reaction caused by urate deposition in the joint capsule, synovial bursa, cartilage, bone, tendon, and other tissues due to hyperuricemia, which is mainly manifested in swelling, pain, and fever of joints and/or tissues around joints [1–3]. Repeated attacks may form tophi, leading to joint deformities, joint damages, and dyskinesia [3]. In recent years, the incidence of GA has been increasing worldwide, seriously affecting the quality of life and work efficiency of patients and causing a heavy medical burden to the society [4, 5]. At present, colchicine, nonsteroidal anti-inflammatory drugs, allopurinol, benzbromarone, febuxostat, etc., are common Western medicines used for treating GA, but they can cause serious adverse reactions and side effects such as renal function damage, abnormal liver function, gastrointestinal diseases, and cardiovascular and cerebrovascular diseases [6]. Interleukin-1 (IL-1) inhibitors are second-line drugs for GA, including direct inhibitors of interleukin-1 beta (IL-1*β*) (canakinumab and gevokizumab), selective inhibitors of IL-1 receptors (anakinra), and IL-1*β* receptor antagonist (rilonacept), but treatment-induced adverse reactions, especially those related to upper respiratory tract infections, abscesses, and gastrointestinal diseases, are still unavoidable [7]. Therefore, it is urgent to further develop new drugs that not only have good curative effects on GA but also have fewer side effects.

Tongfengding capsule (TFDC), which contains 8 kinds of traditional Chinese medicine, including *Gentiana Macrophylla Pall* (Qinjia), *Phellodendri Chinrnsis Cortex* (Huangbo), *Corydalis Rhizoma* (Yanhuosuo), *Radix Paeoniae Rubra* (Chishao), *Cyathulae Radix* (Chuanniuxi), *Smilacis Glabrae Rhixoma* (Tufuling), *Plantaginis Semen* (Cheqianzi), and *Alisma Orientale (Sam.) Juz.* (Zexie), is one of the Chinese patent medicines approved by the National Medical Products Administration (NMPA) of the People's Republic of China and listed in the Chinese Pharmacopoeia (2020) [8]. It can clear away heat and dampness, promote blood circulation, dredge collaterals, and calm pain, which is mainly used for treating gout. The study found that TFDC might inhibit the inflammatory response in treating gout by regulating the arachidonic acid metabolic pathway, especially its active compound apigenin could significantly inhibit the related targets of prostaglandin G/H synthase 2 (PTGS2), tumor necrosis factor-*α* (TNF-*α*), and interleukin-6 (IL-6) in this pathway [9]. Clinically, TFDC could improve blood uric acid levels in patients and reduce adverse reactions such as stomach discomfort and mild diarrhea, so as to better treat GA [10].

IL-1*β*, a key bone marrow-derived proinflammatory cytokine, involves in a variety of autoimmune inflammatory responses and cellular activities including cell proliferation, differentiation, and apoptosis [11]. When IL-1*β* is excessively produced, it can cause atherosclerosis, rheumatoid arthritis, gout, Parkinson's disease, Alzheimer's disease, tumors, and other diseases [12, 13]. In particular, IL-1*β* plays a key role in the occurrence and development of GA [14]. Urate deposition in GA promotes the release of IL-1*β*, which in turn promotes the influx of neutrophils into the joint to trigger joint inflammation [15–18]. Moreover, tophi themselves also have a large number of cells expressing IL-1*β* [15]. IL-1*β* also has been implicated in bone destruction and bone erosion in GA [15]. In view of this, some in-depth studies have revealed that the herbal medicine simiaosan can alleviate the symptoms of GA by regulating the NALP3/IL-1*β* signaling pathway, and that curcumin can suppress the expression of Nod-like receptor 3 (NLRP3) inflammasome to downregulate the level of IL-1*β* via inhibiting the activation of nuclear factor-kappa B (NF-*κ*B) signaling pathway in monosodium urate (MSU)-induced GA [19, 20]. Anyway, there is an urgent need and important clinical value to develop inhibitors targeting IL-1*β* in GA with better efficacy and fewer side effects.

**Figure 1:**
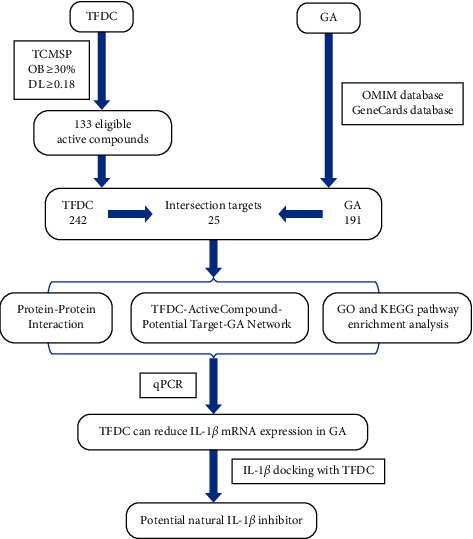
The workflow of IL-1*β* inhibitor prediction in GA.

Network pharmacology, originally proposed by Andrew L. Hopkins in 2007, mainly studies the complex and diverse relationships among drugs, targets, diseases, and pathways, providing new methods for the development of new drugs and new ideas for the treatment of some diseases [21–23]. Molecular docking, developed with the continuous update of computer technology and the rapid increase of structural, chemical, and biological data related to available therapeutic targets, can be used to identify new compounds with therapeutic significance and contribute to drug discovery and development [24, 25]. This study firstly screened the active compounds of TFDC and then analyzed and summarized the possible targets of active compounds in treating GA. Next, the targets of active compounds and the pathways of targets that might play a therapeutic role were investigated by using network pharmacology, Gene Ontology (GO), and Kyoto Encyclopedia of Genes and Genomes (KEGG) pathway enrichment analyses. And then, the therapeutic effect of TFDC on IL-1*β*, an important target in the pathological mechanism of GA, was verified by the experiment. Finally, the optimal active compounds of TFDC docked with IL-1*β* were analyzed by molecular docking technology in order to explore and develop novel optimum compounds that could inhibit IL-1*β* in GA. Taken together, this study aimed to provide theoretical basis and more research ideas for the molecular mechanism of TFDC against GA and discover more natural compounds as IL-1*β* inhibitors that can be used to better treat GA with fewer side effects. The workflow is shown in Figure 1.

## 2. Method and Materials

### 2.1. Identification of Active Compounds and Related Targets in TFDC

The Traditional Chinese Medicine Systems Pharmacology Database and Analysis Platform (TCMSP, https://tcmspw.com/index.php) [26] was used to screen the active compounds and related targets of 8 Chinese herbal medicines in TFDC with the criteria of oral bioavailability (OB) ≥ 30% and drug-likeness (DL) ≥ 0.18. Then, the UniProt database (https://www.uniprot.org/) [27] was used to obtain the gene symbol and UniProt ID of the targets screened by TCMSP with the species selected as “*Homo sapiens*”.

### 2.2. Identification of TFDC Targets Related to GA

The GA-related targets were collected from GeneCards (https://www.genecards.org/) [28] and OMIM (Online Mendelian Inheritance in Man, https://omim.org/search/advanced/geneMap) [29] with the keywords “gouty arthritis”. Then, the gene symbol and UniProt ID of the GA-related targets were obtained from UniProt as well. Potential targets of TFDC for GA were acquired through the Venny 2.1 (https://bioinfogp.cnb.csic.es/tools/venny/) intersection.

### 2.3. Construction of Protein-Protein Interaction (PPI) Network

The TFDC-GA-potential targets were processed by the STRING database (https://string-db.org/) [30] with the species limited to “*Homo sapiens*” and a confidence score of >0.9. Then, the PPI network of TFDC-GA-potential targets was imported into Cytoscape software (version 3.8.0) [31] for network analysis. Nodes represent targets in the PPI network map, and edges represent functional associations between potential targets.

### 2.4. Construction of TFDC-Active Compound-Potential Target-GA Network

To accurately elicit interactions between active compounds and its corresponding GA-related targets of TFDC, a visual network was established through Cytoscape software. Furthermore, the network topology parameters were applied to select the key compounds and targets.

### 2.5. Gene Ontology (GO) and Kyoto Encyclopedia of Genes and Genomes (KEGG) Pathway Enrichment Analysis

The packages “org.Hs.eg.db”, “DOSE”, “enrichplot”, “clusterProfiler” [32], and “ggplot2” in R software (version 4.1.1 for Windows) were used to carry out GO and KEGG pathway enrichment analyses and visualization for TFDC-GA-potential targets with *P* < 0.05.

### 2.6. Cell Culture and Treatment

Human monocytic leukemia THP-1 cell was purchased from Procell Life Science & Technology Co., Ltd (Wuhan, Hubei Province, China). Cells were cultured in RPMI-1640 medium supplemented with 10% fetal bovine serum (Gibco, USA), 100  U/mL penicillin, and 100 *μ*g/mL streptomycin (Gibco, USA). They were maintained in an incubator at 37°C with 5% CO_2_ and saturated humidity, and the culture medium was replaced with complete culture medium every 2 or 3 days. The cells were treated with phorbol-12-myristate-13-acetate (PMA) (100 nM) for 3 h to induce their differentiation into resting M0 macrophage. A fresh complete culture medium was added and cultured for 24 h after washing the cells with phosphate-buffered saline (PBS). Differentiated THP-1 cells were then stimulated with MSU (50, 100, and 200 *μ*g/mL) with optimal concentration and treated with TFDC at final concentrations of 25, 50, 75, 100, and 125 *μ*g/mL as well as colchicine (positive drug, 2 *μ*g/mL) simultaneously at 37°C for 24 h [33].

### 2.7. Cell Viability Analysis

THP-1 cells were harvested during the logarithmic growth phase and seeded into a 96-well plate at a density of 1 × 10^6^ cells/well with a final volume of 100 *μ*L. After treatment, 10 *μ*L of CCK-8 was added to each well, and the plates were then incubated for additional 2 h. The absorbance at 450 nm was measured using a microplate reader. The experiments were performed in quadruplicate and repeated at least three times.

### 2.8. Quantitative Real-Time PCR (qPCR) Analysis

Total RNA was extracted from cells in 12-well plates, and cDNA was prepared according to the manufacturer's protocol. qPCR was amplified and measured on the BIO-RAD CFX96TM (BIO-RAD, USA) in the presence of SYBR Green. Then, the fluorescence values were collected, and a melting curve analysis was performed. The experiments were conducted at least three times. The sequences of IL-1*β* and *β*-actin primers are, respectively, as follow: Forward: CTCGCCAGTGAAATGATGGCT, Reverse: GTGGTGGTCGGAGATTCGTAG; and Forward: TGGCACCCAGCACAATGAA, Reverse: CTAAGTCATAGTCCGCCTAGAAGCA. The relative quantifications of genes expression were calculated using the 2^−ΔΔCq^ method with *β*-actin served as a normalization control.

### 2.9. Verification between Active Compounds and Key Targets by Molecular Docking

Active compounds selected from the TFDC-active compound-potential target-GA network were molecularly docked with IL-1*β* receptor. Before performing the docking progress, the 2D structures of active compounds were acquired from the PubChem database (https://pubchem.ncbi.nlm.nih.gov/) [34] and optimized to save as 3D structures by Chem3D software. The 3D protein structure of IL-1*β* receptor was acquired from the RCSB PDB database (https://www.rcsb.org/) [35]. Then, AutoDockTools software was used to process molecular structures followed by AutoDockVina software used for molecular docking. Last, visualization of the docking results was carried out by PyMOL and Discovery Studio software.

### 2.10. Statistical Analysis

Data were expressed as the mean ± standard deviation (SD). Intergroup differences were determined using one-way ANOVA followed by Tukey's comparison tests. *P* < 0.05 was considered statistically significant. All data were processed using GraphPad Prism 8.3.0 software.

## 3. Results

### 3.1. Acquirement of Active Compounds in TFDC

Based on the criteria of OB ≥ 30% and DL ≥ 0.18, a total of 155 (after removing duplication: 133) eligible active compounds of TFDC were screened, of which 9 were from Cheqianzi (CQZ), 29 were from Chishao (CS), 4 were from Chuanniuxi (CNX), 37 were from Huangbo (HB), 2 were from Qinjia (QJ), 15 were from Tufuling (TFL), 49 were from Yanhuosuo (YHS), and 10 were from Zexie (ZX). The characteristics of all the screened out eligible active compounds in the TFDC are shown in Table 1.

### 3.2. Acquirement of the Potential Therapeutic Targets and Analysis of the PPI Network

**Figure 2:**
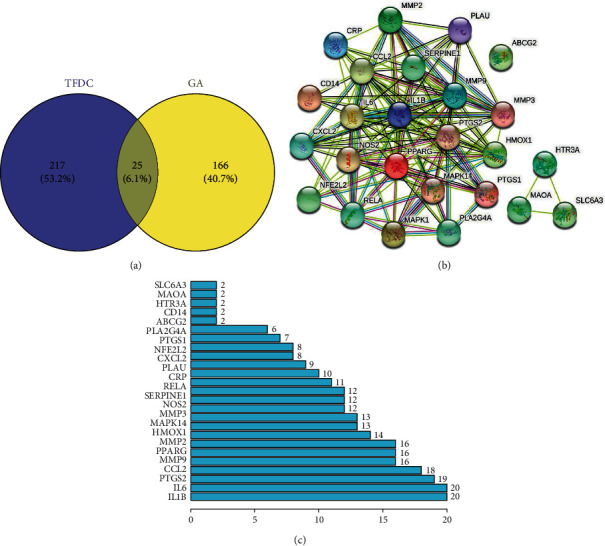
Potential therapeutic targets and PPI network map of TFDC for GA. (a) The Venny results of potential therapeutic targets of TFDC for GA. (b) The PPI network map of 25 targets. (c) Count and list of the above targets of PPI network map.

A total of 207 GA-related targets were obtained from GeneCards and OMIM databases. After removing duplicate targets, 191 GA-related targets in total were collected at last. Similarly, 242 unrepeated targets of 133 active compounds in the TFDC were obtained from TCMSP database. The Venny 2.1 platform was used to intersect the GA-related and TFDC-related targets. 25 potential therapeutic targets were obtained (Figure 2(a)) (Table 2). Sequentially, the above targets were imported into the STRING database to obtain the PPI network (Figure 2(b)). The above targets of the PPI network results were identified and listed by the R package “Venn Diagram” (Figure 2(c)). These genes include mitogen-activated protein kinase 14 (MAPK14), interleukin-1 beta (IL1B, IL-1*β*), prostaglandin G/H synthase 1 (PTGS1), peroxisome proliferator activated receptor gamma (PPARG), transcription factor p65 (RELA), and matrix metalloproteinase-9 (MMP9). Among them, IL-1*β* is at the core because it is most associated with other targets in the PPI network, which indicates that IL-1*β* plays a significant role in the occurrence and development of GA.

### 3.3. Construction and Analysis of the TFDC-Active Compound-Potential Target-GA Network

**Figure 3:**
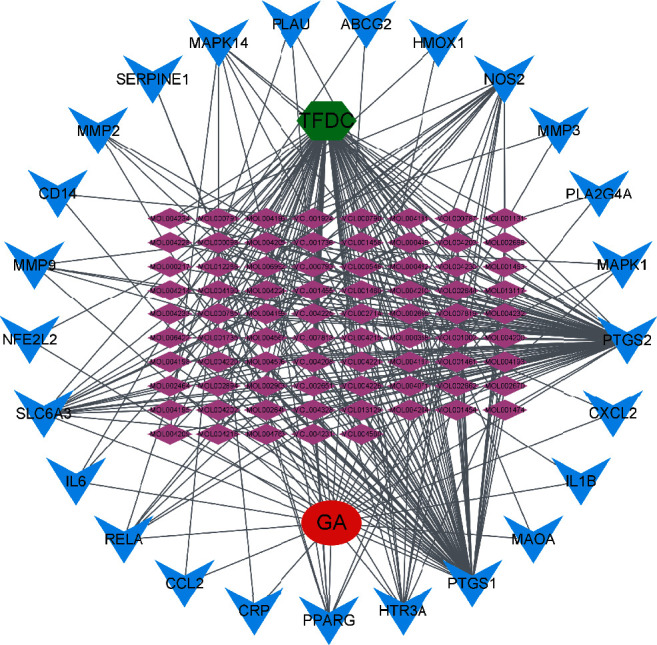
The TFDC-active compound-potential target-GA network. The green node represents TFDC. The red node represents GA. Purple nodes represent active compounds. Blue nodes represent targets. Gray lines represent interconnections between nodes and nodes.

The potential targets and their corresponding eligible active compounds were entered into the Cytoscape software to obtain a TFDC-active compound-potential target-GA network (Figure 3). A total of 104 nodes and 332 edges were obtained from the TFDC-active compound-potential target-GA network. The results of network topology analysis showed that the top 10 targets, namely, prostaglandin G/H synthase 2 (PTGS2), PTGS1, nitric oxide synthase, inducible (NOS2), sodium-dependent dopamine transporter (SLC6A3), 5-hydroxytryptamine receptor 3A (HTR3A), PPARG, MAPK14, RELA, MMP9, and 72 kDa type IV collagenase (MMP2), have a higher degree, which indicates that they play important roles in this interaction network (degree ≥ 4) (Table 3). Besides, the top 7 active compounds, namely, quercetin, stigmasterol, betavulgarin, rutaecarpine, naringenin, dihydrochelerythrine, and dihydrosanguinarine, also have a higher degree, which have remarkable significance in the network (degree ≥ 6) (Table 4).

### 3.4. GO and KEGG Pathway Enrichment Analysis

**Figure 4:**
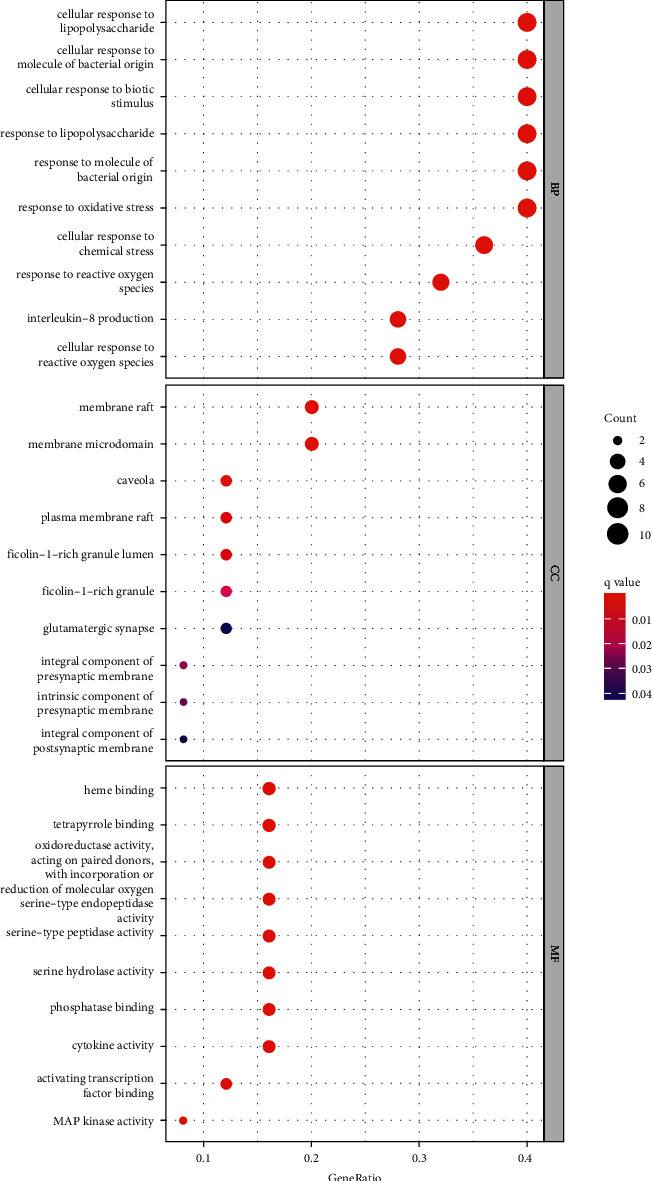
GO enrichment analysis of potential targets of TFDC in GA.

**Figure 5:**
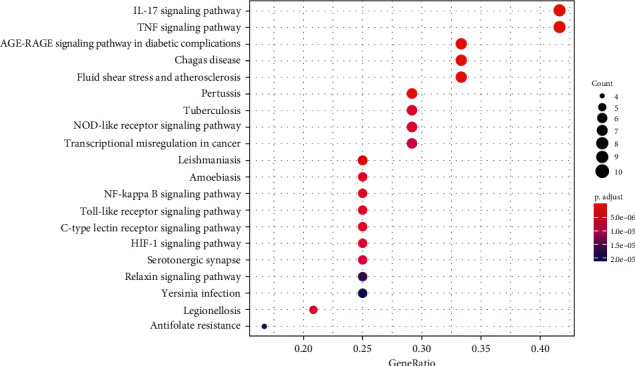
KEGG pathway enrichment analysis of potential targets of TFDC in GA.

**Figure 6:**
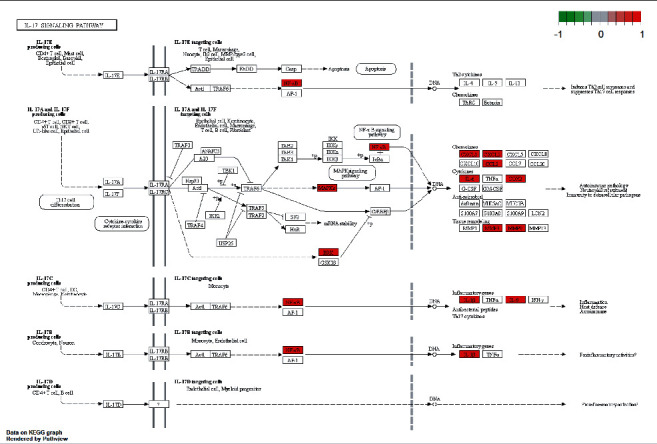
The IL-17 signaling pathway of potential targets of TFDC in GA. Arrows indicate upstream and downstream relationships between targets. The red color represents TFDC-related targets in the network.

**Figure 7:**
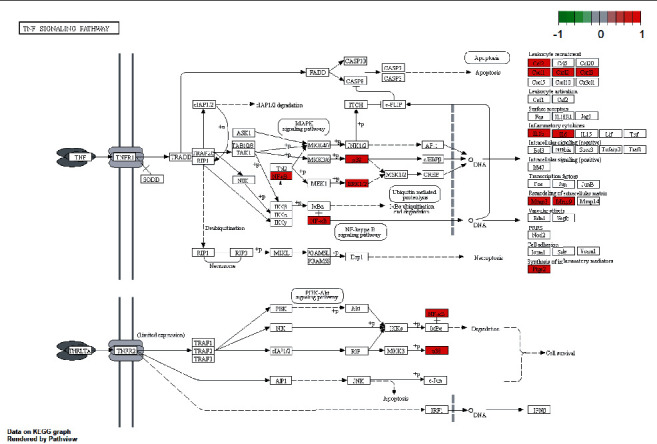
The TNF signaling pathway of potential targets of TFDC in GA. Arrows indicate upstream and downstream relationships between targets. The red color represents TFDC-related targets in the network.

In order to elucidate the biological mechanisms of TFDC against GA, GO and KEGG pathway enrichment analyses were performed by using clusterProfiler in R. The 25 potential targets of TFDC for treating GA were input into the R, and a total of 1209 GO terms with remarkable significance were obtained, including 1100 biological process (BP) terms, 95 molecular function (MF) terms, and 14 cellular component (CC) terms. The results of GO enrichment analysis mainly include cellular response to lipopolysaccharide, cellular response to molecule of bacterial origin, membrane raft, membrane microdomain, heme binding, tetrapyrrole binding, etc. (Figure 4). Additionally, a total of 93 enriched KEGG pathways were obtained, mainly including interleukin-17 (IL-17) signaling pathway, tumor necrosis factor (TNF) signaling pathway, NOD-like receptor signaling pathway, NF-*κ*B signaling pathway, Toll-like receptor signaling pathway, etc. (Figure 5). What's more, the vital IL-17 signaling pathway and TNF signaling pathway are shown in Figure 6 and Figure 7, respectively.

### 3.5. Effect of TFDC on Cell Viability

**Figure 8:**
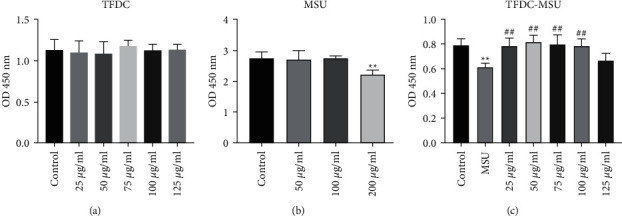
Effects of TFDC on MSU-induced THP-1 cell viability. (a) THP-1 cells were exposed to TFDC at various concentrations for 24 h. (b) THP-1 cells were exposed to MSU at various concentrations for 24 h. (c) Protective effects of TFDC on the viabilities of MSU-induced THP-1 cells. Cell viability was assessed by CCK-8 assay and expressed relative to untreated control cells. ^*∗∗*^*P* < 0.01 versus control group. ^##^*P* < 0.01 versus MSU group.

The effects of TFDC and MSU on THP-1 cells were respectively evaluated with a CCK-8 assay and are shown in Figure 8(a) and 8(b). According to the result of OD value, TFDC at final concentrations of 25, 50, 75, 100, and 125 *μ*g/mL had little effect on the viabilities of THP-1 cells (*P* > 0.05). THP-1 cells stimulated with MSU (50 and 100 *μ*g/mL) showed insignificant differences compared with the control group (*P* > 0.05), but that with 200 *μ*g/mL MSU obviously decreased the viabilities of THP-1 cell (*P* < 0.01). Based on the above results, 200 *μ*g/mL was the optimum induction dosage in further experiments. For anti-inflammatory activity, MSU-induced THP-1 cells treated with TFDC among 25, 50, 75, 100, and 125 *μ*g/mL exhibited different degrees of protection (Figure 8(c)). 50 *μ*g/mL TFDC had the best protective effect on the viabilities of MSU-induced THP-1 cells, and the concentration of TFDC was defined to 50 *μ*g/mL for qPCR verification.

### 3.6. Effect of TFDC on IL-1*β*

**Figure 9:**
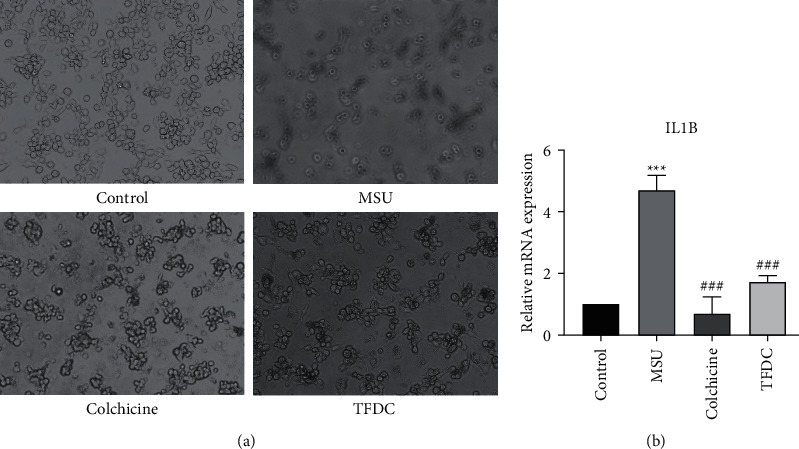
TFDC protects THP-1 cells against MSU-induced inflammation by affecting the expression of IL-1*β*. (a) Effects of TFDC on MSU-induced THP-1 cells. (b) Statistical analysis of the effect of TFDC on the mRNA expression level of IL-1*β*. Data are presented as the mean ± SD (*n* = 3). ^*∗∗∗*^*P* < 0.001 versus control group. ^###^*P* < 0.001 versus MSU group.

By observation under microscope, the cell density of the MSU group clearly reduced compared with that of the control group, while the cell densities of the colchicine group and the TFDC group both significantly exceeded that of the MSU group (Figure 9(a)). In order to further validate whether TFDC could inhibit IL-1*β* in GA against inflammation, mRNA expression of IL-1*β* was examined by qPCR analysis. The IL-1*β* mRNA expression in the MSU group was remarkably higher than that in the control group (*P* < 0.001) (Figure 9(b)). Compared with the MSU group, the IL-1*β* mRNA expressions in the colchicine group and the TFDC group were separately apparently decreased (*P* < 0.001) (Figure 9(b)). The results suggested that the anti-inflammation of TFDC on GA was associated with inhibition of IL-1*β* mRNA expression.

### 3.7. Molecular Docking Results of Active Compounds and IL-1*β*

**Figure 10:**
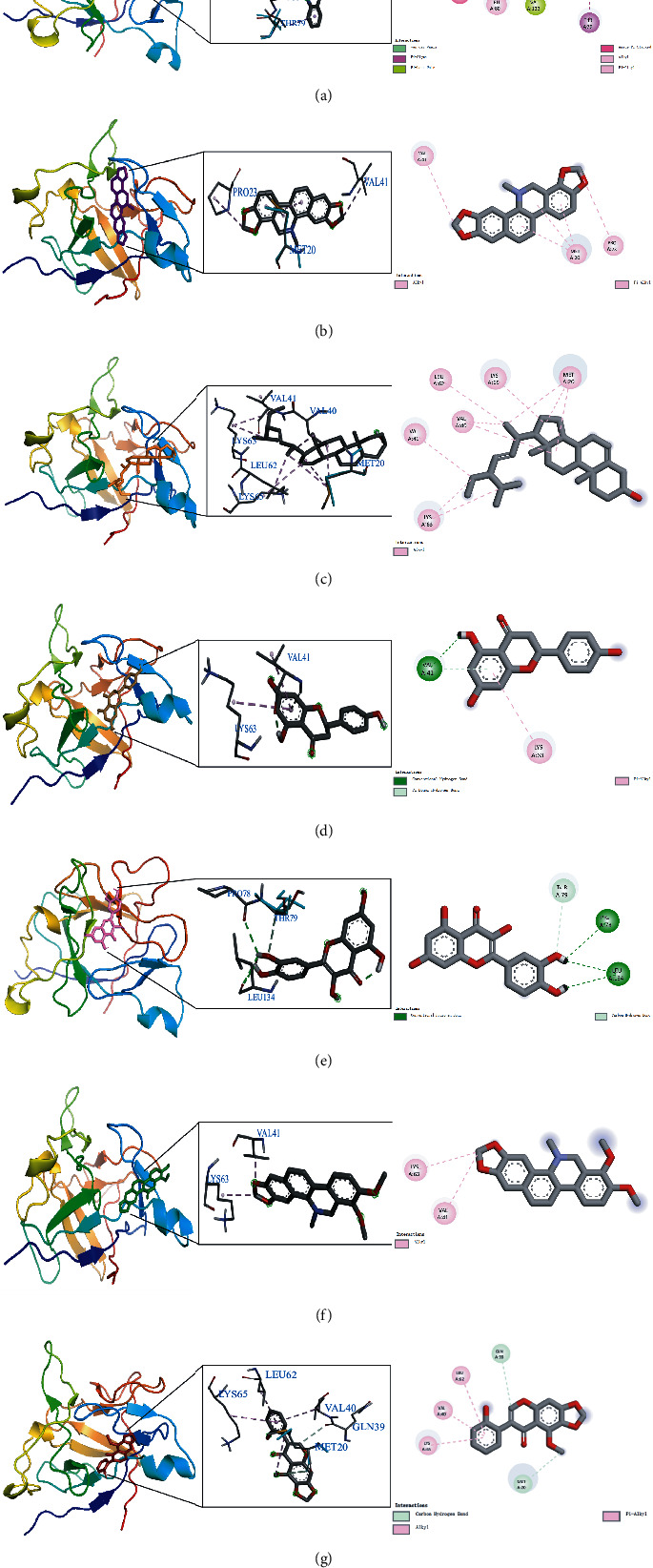
The diagram of the binding of rutaecarpine (a), dihydrosanguinarine (b), stigmasterol (c), naringenin (d), quercetin (e), dihydrochelerythrine (f), and betavulgarin (g) with IL-1*β*.

The top 7 high-degree active compounds from the TFDC-active compound-potential target-GA network bind IL-1*β* to varying degrees (Table 5). The lower the binding energy, the stronger and more stable the interaction between the active compounds and receptor. The binding energy of rutaecarpine, dihydrosanguinarine, stigmasterol, naringenin, quercetin, dihydrochelerythrine, and betavulgarin increased sequentially, indicating that rutaecarpine had the strongest and most stable binding affinity toward IL-1*β*. The diagrams of the binding of rutaecarpine, dihydrosanguinarine, stigmasterol, naringenin, quercetin, dihydrochelerythrine, and betavulgarin to IL-1*β* are shown in Figure 10. As shown in the figure, for example, rutaecarpine formed van der Waals with IL-1*β* protein structure 5R8Q amino acid residue A chain LEU82, formed *π*-*σ* interaction with amino acid residue A chain THR79, formed *π*-lone pair interaction with amino acid residue A chain VAL132, formed amide-*π* stacked interaction with amino acid residue A chain GLN81, and formed alkyl and *π*-alkyl interactions with amino acid residue A chain PRO131 and LEU80. These interactions reduced the energy required for binding, which increased the affinities between the active compounds and IL-1*β* protein structure 5R8Q to make them easier to bind.

## 4. Discussion

GA, as a chronic inflammatory disease caused by the deposition of urate in the joints, is mainly characterized by redness, swelling, heat, and pain in the affected joints [36]. Repeated attacks can form tophi, leading to bone erosion, destroying the joints, and causing movement obstacles or severe disability, which have a serious negative impact on the quality of life of patients and bring huge psychological and economic burden to patients [37, 38]. It has been clear that the pathology of GA involves the production and release of the inflammatory factor IL-1*β*, and the progression of GA can be mediated by IL-1*β* [39, 40]. In this study, the PPI network revealed that the potential targets of TFDC for GA are interacting rather than isolated, resulting in a synergistic therapeutic effect between the targets. Moreover, the network also showed that IL-1*β* was the most associated target with other targets, indicating that IL-1*β* was the most critical target in TFDC treating GA. Based on this, the qPCR experiment also confirmed that TFDC could obviously inhibit IL-1*β*, playing an important role in the occurrence and development of GA, which provided strong experimental evidence support for confirming that TFDC could indeed treat GA.

In addition, in this study, the analysis results of the TFDC-active compound-potential target-GA network also found that these seven active compounds quercetin, stigmasterol, betavulgarin, rutaecarpine, naringenin, dihydrochelerythrine, and dihydrosanguinarine and these 10 targets PTGS2, PTGS1, NOS2, SLC6A3, HTR3A, PPARG, MAPK14, RELA, MMP9, and MMP2 played important roles in treating GA, which are worthy of further research in the future. Studies found that quercetin had a significant joint protective effect in gouty arthritis and, in the mouse model of gouty arthritis induced by monosodium urate crystals (MSU), exerted analgesic and anti-inflammatory effects in a naloxone-sensitive manner [41, 42]. In MSU-induced rat models with GA, quercetin alleviated edema, reduced the histological signs of acute inflammation, inhibited leukocyte recruitment, and reduced the level of chemokines in a dose-dependent manner, which exhibited a strong anti-inflammatory effect [43]. Previous studies pointed out that stigmasterol could reduce the serum uric acid level of hyperuricemia mice by inhibiting the activity of hepatic xanthine oxidase and significantly reduce the claw edema caused by MSU, which could be a promising drug for treating gouty arthritis, hyperuricemia, and inflammation [44]. At present, there are no research reports on treating gouty arthritis with betavulgarin, rutaecarpine, naringenin, dihydrochelerythrine, and dihydrosanguinarine, suggesting that these five active compounds can be used as promising candidate drugs for follow-up experimental research to verify the efficacy of GA. Once experimental studies confirm that these active compounds can be effective in the GA model, they will be essential discoveries and provide more possibilities for drug development of IL-1*β* inhibitors. Not only that, the verification results of molecular docking showed that rutaecarpine was confirmed to be the active compound that most easily bound to the IL-1*β* receptor, which is a natural IL-1*β* inhibitor with great therapeutic potential and potential research value. Moreover, the remaining 6 active compounds were also relatively easy to bind to the IL-1*β* receptor, and all had good affinities. Many studies showed that inhibition of PTGS2 (i.e., COX-2), NOS2, MAPK14, MMP9, MMP2, and activation of PPARG could improve inflammation in GA models, suggesting that these targets had potential importance in the pathogenesis of GA [45–51].

GO enrichment analysis of potential therapeutic targets revealed that they mainly involved biological functions such as cellular response to lipopolysaccharide, cellular response to molecule of bacterial origin, membrane raft, membrane microdomain, heme binding, and tetrapyrrole binding. Meanwhile, the main signaling pathways enriched for potential therapeutic targets were IL-17 signaling pathway, TNF signaling pathway, NOD-like receptor signaling pathway, NF-*κ*B signaling pathway, Toll-like receptor signaling pathway, etc. These biological functions and signaling pathways were related to the occurrence and development of GA, which might be the mechanism of TFDC in the treatment of GA. In particular, IL-17 signaling pathway and TNF signaling pathway played an extremely vital role in exploring the therapeutic mechanisms obtained by the analysis. IL-17, an important proinflammatory cytokine associated with several autoimmune diseases, significantly increased in the serum of patients with GA in the early stage of acute exacerbations, positively correlated with disease activity, and also correlated with the IL-1*β* level in serum [52]. In the GA rat model, the ratio of Treg/Th17 in the spleen decreased with the occurrence of joint inflammation, suggesting that the imbalance of Treg/Th17 might be related to the pathogenesis of acute gouty arthritis [53]. The GA mouse model could produce Th17 cells and their related inflammatory cell chemokines such as IL-17, and the use of neutralizing antibodies against IL-17 could reduce joint swelling and leukocyte infiltration into inflammatory sites [54]. TNF-*α* is a member of the TNF family and participates in systemic inflammation. It was confirmed that IL-1*β*, TNF-*α*, IL-6, interleukin-8 (IL-8), and interleukin-4 (IL-4) concentrations in the serum of GA patients increased significantly, which might be related to the pathogenesis of GA [55]. It was found that high expression of Cyr61 protein could induce MSU-stimulated rat synovial cells to produce many inflammatory cytokines such as IL-1*β*, TNF-*α*, and IL-6 [56]. And TNF-*α* could promote the secretion of IL-1*β* in MSU-induced human neutrophils [57]. Besides, many studies showed that regulating NOD-like receptor signaling pathway, NF-*κ*B signaling pathway, Toll-like receptor signaling pathway, and their representative targets such as NOD-, LRR-, and pyrin domain-containing protein 3 (NLRP3) inflammasome, Toll-like receptor-4 (TLR4), Toll-like receptor-2 (TLR2), IKK*α*, I*κ*B*α*, and NF-*κ*B, could effectively improve the joint inflammatory response of the GA model [20, 58–61]. These all indicate that the above signaling pathways are involved in the pathogenesis of GA and play a significant role in the development of the disease, by regulating which the disease progression of GA can be effectively controlled. At the same time, these all suggest that TFDC treating GA is accomplished through multifunction and multichannel synergy, and some functions and pathways are more important, which can be taken as a basis and reference for the direction of further research.

## 5. Conclusion

In conclusion, the TFDC has obvious advantages and significant efficacy in the treatment of GA, which is consistent with the published results of relevant clinical and experimental studies. In this study, the network pharmacology method was used to investigate the biological functions and signaling pathways of the targets of TFDC's active compounds in the treatment of GA. Simultaneously, the experiment clearly verified that the TFDC could significantly inhibit the key target IL-1*β* in GA. In addition, rutaecarpine, one of the active compounds in TFDC, had the best binding activity with IL-1*β* by the molecular docking method, which could be investigated as the most suitable natural IL-1*β* inhibitor. These findings have provided new clues and ideas for further research on the molecular biological mechanism of TFDC in the treatment of GA and offered a practical and reliable theoretical basis for the clinical treatment of GA by TFDC.

## Figures and Tables

**Table 1:** Characteristics of eligible active compounds in TFDC.

No.	Molecule ID	Molecule name	Molecular weight	OB (%)	DL	Herbs
1	MOL000791	Bicuculline	367.38	69.67	0.88	YHS
2	MOL002668	Worenine	334.37	45.83	0.87	HB
3	MOL001463	Dihydrosanguinarine	333.36	59.31	0.86	YHS
4	MOL004225	Pseudocoptisine	320.34	38.97	0.86	YHS
5	MOL001474	Sanguinarine	332.35	37.81	0.86	YHS
6	MOL004231	Tetrahydrocorysamine	337.40	34.17	0.86	YHS
7	MOL001458	Coptisine	320.34	30.67	0.86	HB, YHS
8	MOL004230	Stylopine	323.37	48.25	0.85	YHS
9	MOL004198	18797-79-0	367.43	46.06	0.85	YHS
10	MOL000787	Fumarine	353.40	59.26	0.83	HB, YHS
11	MOL004226	24240-05-9	353.40	53.75	0.83	YHS
12	MOL002673	Hispidone	472.78	36.18	0.83	HB
13	MOL000856	Alisol C monoacetate	514.77	33.06	0.83	ZX
14	MOL007835	Orobanchoside_qt	476.47	55.99	0.82	CQZ
15	MOL002660	Niloticin	456.78	41.41	0.82	HB
16	MOL000853	Alisol B	444.72	36.76	0.82	ZX
17	MOL006392	Dihydroniloticin	458.80	36.43	0.82	HB
18	MOL006413	Phellochin	488.83	35.41	0.82	HB
19	MOL000830	Alisol B	472.78	34.47	0.82	ZX
20	MOL002636	Kihadalactone A	512.70	34.21	0.82	HB
21	MOL000854	Alisol C	486.76	32.70	0.82	ZX
22	MOL000832	Alisol B 23-acetate	446.74	32.52	0.82	ZX
23	MOL000546	Diosgenin	414.69	80.88	0.81	TFL
24	MOL004202	Dehydrocavidine	351.43	38.99	0.81	YHS
25	MOL002656	Dihydroniloticin	458.80	36.43	0.81	HB
26	MOL002670	Cavidine	353.45	35.64	0.81	HB, YHS
27	MOL000831	Alisol B monoacetate	514.82	35.58	0.81	ZX
28	MOL000862	[(1S,3R)-1-[(2R)-3,3-dimethyloxiran-2-yl]-3-[(5R,8S,9S,10S,11S,14R)-11-hydroxy-4,4,8,10,14-pentamethyl-3-oxo-1,2,5,6,7,9,11,12,15,16-decahydrocyclopenta[a]phenanthren-17-yl]butyl] acetate	514.82	35.58	0.81	ZX
29	MOL001461	Dihydrochelerythrine	349.41	32.73	0.81	YHS
30	MOL001921	Lactiflorin	462.49	49.12	0.80	CS
31	MOL004190	(−)-Alpha-N-methylcanadine	354.46	45.06	0.80	YHS
32	MOL001924	Paeoniflorin	480.51	53.87	0.79	CS
33	MOL004228	Saulatine	396.47	42.74	0.79	YHS
34	MOL006401	Melianone	470.76	40.53	0.78	HB
35	MOL001454	Berberine	336.39	36.86	0.78	HB, YHS
36	MOL002666	Chelerythrine	332.37	34.18	0.78	HB
37	MOL002903	(R)-canadine	339.42	55.37	0.77	YHS
38	MOL001455	(S)-canadine	339.42	53.83	0.77	HB
39	MOL013352	Obacunone	454.56	43.29	0.77	HB
40	MOL000849	16*β*-methoxyalisol B monoacetate	544.85	32.43	0.77	ZX
41	MOL007004	Albiflorin	480.51	30.25	0.77	CS
42	MOL000449	Stigmasterol	412.77	43.83	0.76	CS, HB, TFL, YHS
43	MOL004355	Spinasterol	412.77	42.98	0.76	CS
44	MOL001663	(4aS,6aR,6aS,6bR,8aR,10R,12aR,14bS)-10-hydroxy-2,2,6a,6b,9,9,12a-heptamethyl-1,3,4,5,6,6a,7,8,8a,10,11,12,13,14b-tetradecahydropicene-4a-carboxylic acid	456.78	32.03	0.76	CQZ
45	MOL002776	Baicalin	446.39	40.12	0.75	CS
46	MOL006999	Stigmast-7-en-3-ol	414.79	37.42	0.75	CS
47	MOL002643	Delta 7-stigmastenol	414.79	37.42	0.75	HB
48	MOL000359	Sitosterol	414.79	36.91	0.75	CQZ, CS, QJ, TFL, YHS, ZX
49	MOL005869	Daucosterol_qt	414.79	36.91	0.75	CQZ
50	MOL000358	Beta-sitosterol	414.79	36.91	0.75	CS, CNX, HB, QJ, TFL
51	MOL001771	Poriferast-5-en-3beta-ol	414.79	36.91	0.75	HB
52	MOL013119	Enhydrin	464.51	40.56	0.74	TFL
53	MOL013118	Neoastilbin	450.43	40.54	0.74	TFL
54	MOL004575	Astilbin	450.43	36.46	0.74	TFL
55	MOL004234	2,3,9,10-Tetramethoxy-13-methyl-5,6-dihydroisoquinolino[2,1-b]isoquinolin-8-one	381.46	76.77	0.73	YHS
56	MOL006422	Thalifendine	322.36	44.41	0.73	HB
57	MOL002894	Berberrubine	322.36	35.74	0.73	HB
58	MOL001460	Cryptopin	369.45	78.74	0.72	YHS
59	MOL004210	(1S,8′R)-6,7-dimethoxy-2-methylspiro[3,4-dihydroisoquinoline-1,7′-6,8-dihydrocyclopenta[g][1,3]benzodioxole]-8′-ol	369.45	43.95	0.72	YHS
60	MOL005043	Campest-5-en-3beta-ol	400.76	37.58	0.71	CS
61	MOL005438	Campesterol	400.76	37.58	0.71	HB
62	MOL004224	Pontevedrine	381.41	30.28	0.71	YHS
63	MOL004567	Isoengelitin	434.43	34.65	0.70	TFL
64	MOL002659	Kihadanin A	486.56	31.60	0.70	HB
65	MOL004191	Capaurine	371.47	62.91	0.69	YHS
66	MOL000793	C09367	325.39	47.54	0.69	YHS
67	MOL002671	Candletoxin A	608.79	31.81	0.69	HB
68	MOL004195	Corydaline	369.50	65.84	0.68	YHS
69	MOL004204	Dehydrocorydaline	366.47	41.98	0.68	YHS
70	MOL004214	Isocorybulbine	368.51	40.18	0.66	YHS
71	MOL000785	Palmatine	352.44	64.60	0.65	HB, YHS
72	MOL000762	Palmidin A	510.52	35.36	0.65	HB
73	MOL004071	Hyndarin	355.47	73.94	0.64	YHS
74	MOL004203	Dehydrocorybulbine	352.44	46.97	0.63	YHS
75	MOL004216	13-Methylpalmatrubine	352.44	40.97	0.63	YHS
76	MOL002672	Hericenone H	580.88	39.00	0.63	HB
77	MOL004209	13-Methyldehydrocorydalmine	352.44	35.94	0.63	YHS
78	MOL002662	Rutaecarpine	287.34	40.30	0.60	HB
79	MOL004199	Corynoloxine	365.41	38.12	0.60	YHS
80	MOL004196	Corydalmine	340.45	52.50	0.59	YHS
81	MOL004205	Dehydrocorydalmine	338.41	43.90	0.59	YHS
82	MOL000790	Isocorypalmine	341.44	35.77	0.59	HB, YHS
83	MOL004220	N-methyllaurotetanine	341.44	41.62	0.56	YHS
84	MOL004233	ST057701	341.44	31.87	0.56	YHS
85	MOL004221	Norglaucing	341.44	30.35	0.56	YHS
86	MOL004197	Corydine	341.44	37.16	0.55	YHS
87	MOL004193	Clarkeanidine	327.41	86.65	0.54	YHS
88	MOL004208	Demethylcorydalmatine	327.41	38.99	0.54	YHS
89	MOL000217	(S)-scoulerine	327.41	32.28	0.54	YHS
90	MOL007003	Benzoylpaeoniflorin	584.62	31.14	0.54	CS
91	MOL007025	Isobenzoylpaeoniflorin	584.62	31.14	0.54	CS
92	MOL004763	Izoteolin	327.41	39.53	0.51	YHS
93	MOL012298	Rubrosterone	334.45	32.69	0.47	CNX
94	MOL007014	8-Debenzoylpaeonidanin	390.43	31.74	0.45	CS
95	MOL004200	Methyl-[2-(3,4,6,7-tetramethoxy-1-phenanthryl)ethyl]amine	355.47	61.15	0.44	YHS
96	MOL007008	4-Ethyl-paeoniflorin_qt	332.38	56.87	0.44	CS
97	MOL002641	Phellavin_qt	374.42	35.86	0.44	HB
98	MOL007012	4-o-Methyl-paeoniflorin_qt	332.38	56.70	0.43	CS
99	MOL001002	Ellagic acid	302.20	43.06	0.43	CS
100	MOL001925	Paeoniflorin_qt	318.35	68.18	0.40	CS
101	MOL002651	Dehydrotanshinone II A	292.35	43.76	0.40	HB
102	MOL012286	Betavulgarin	312.29	68.75	0.39	CNX
103	MOL001131	Phellamurin_qt	356.40	56.60	0.39	HB
104	MOL001918	Paeoniflorgenone	318.35	87.59	0.37	CS
105	MOL007016	Paeoniflorigenone	318.35	65.33	0.37	CS
106	MOL006996	1-o-Beta-d-glucopyranosylpaeonisuffrone_qt	332.38	65.08	0.35	CS
107	MOL004232	Tetrahydroprotopapaverine	329.43	57.28	0.33	YHS
108	MOL007005	Albiflorin_qt	318.35	48.70	0.33	CS
109	MOL007018	9-Ethyl-neo-paeoniaflorin A_qt	334.40	64.42	0.30	CS
110	MOL006992	(2R,3R)-4-methoxyl-distylin	318.30	59.98	0.30	CS
111	MOL002464	1-Monolinolein	354.59	37.18	0.30	ZX
112	MOL006994	1-o-Beta-d-glucopyranosyl-8-o-benzoylpaeonisuffrone_qt	302.35	36.01	0.30	CS
113	MOL007813	Dihydrotricetin	304.27	58.12	0.28	CQZ
114	MOL000098	Quercetin	302.25	46.43	0.28	CQZ, CNX, HB, TLF, YHS
115	MOL002644	Phellopterin	300.33	40.19	0.28	HB
116	MOL013117	4,7-Dihydroxy-5-methoxyl-6-methyl-8-formyl-flavan	314.36	37.03	0.28	TFL
117	MOL007819	Hypolaetin	302.25	33.24	0.28	CQZ
118	MOL004580	cis-Dihydroquercetin	304.27	66.44	0.27	TFL
119	MOL013129	(2R,3R)-2-(3,5-dihydroxyphenyl)-3,5,7-trihydroxychroman-4-one	304.27	63.17	0.27	TFL
120	MOL001736	(−)-Taxifolin	304.27	60.51	0.27	TFL
121	MOL004576	Taxifolin	304.27	57.84	0.27	TFL
122	MOL001735	Dinatin	300.28	30.97	0.27	CQZ
123	MOL006990	(1S,2S,4R)-trans-2-hydroxy-1,8-cineole-B-D-glucopyranoside	332.44	30.25	0.27	CS
124	MOL004215	Leonticine	327.46	45.79	0.26	YHS
125	MOL002652	delta7-Dehydrosophoramine	242.35	54.45	0.25	HB
126	MOL000492	(+)-Catechin	290.29	54.83	0.24	CS
127	MOL007836	Plantaginin_qt	288.27	54.04	0.24	CQZ
128	MOL007022	Evofolin B	318.35	64.74	0.22	CS
129	MOL004328	Naringenin	272.27	59.29	0.21	TFL
130	MOL002714	Baicalein	270.25	33.52	0.21	CS
131	MOL002663	Skimmianin	259.28	40.14	0.20	HB
132	MOL000622	Magnograndiolide	266.37	63.71	0.19	HB
133	MOL002883	Ethyl oleate (NF)	310.58	32.40	0.19	CS

OB : oral bioavailability; DL : drug-likeness; YHS : Yanhuosuo; HB : Huangbo; ZX : Zexie; CQZ : Cheqianzi; TFL : Tufuling; CS : Chishao; CNX : Chuanniuxi; QJ : Qinjia.

**Table 2:** 25 potential therapeutic targets of TFDC for GA.

No.	Target	Symbol	Entrez ID
1	72 kDa type IV collagenase	MMP2	4,313
2	Plasminogen activator inhibitor 1	SERPINE1	5,054
3	Mitogen-activated protein kinase 14	MAPK14	1,432
4	Urokinase-type plasminogen activator	PLAU	5,328
5	ATP-binding cassette sub-family G member 2	ABCG2	9,429
6	Heme oxygenase 1	HMOX1	3,162
7	Nitric oxide synthase, inducible	NOS2	4,843
8	Stromelysin-1	MMP3	4,314
9	Cytosolic phospholipase A2	PLA2G4A	5,321
10	Mitogen-activated protein kinase 1	MAPK1	5,594
11	Prostaglandin G/H synthase 2	PTGS2	5,743
12	C-X-C motif chemokine 2	CXCL2	2,920
13	Interleukin-1 beta	IL1B	3,553
14	Amine oxidase [flavin-containing] A	MAOA	4,128
15	Prostaglandin G/H synthase 1	PTGS1	5,742
16	5-Hydroxytryptamine receptor 3A	HTR3A	3,359
17	Peroxisome proliferator activated receptor gamma	PPARG	5,468
18	C-reactive protein	CRP	1,401
19	C–C motif chemokine 2	CCL2	6,347
20	Transcription factor p65	RELA	5,970
21	Interleukin-6	IL6	3,569
22	Sodium-dependent dopamine transporter	SLC6A3	6,531
23	Nuclear factor erythroid 2-related factor 2	NFE2L2	4,780
24	Matrix metalloproteinase-9	MMP9	4,318
25	Monocyte differentiation antigen CD14	CD14	929

**Table 3:** Top 10 high-degree targets in the network.

No.	Target	Symbol	Degree
1	Prostaglandin G/H synthase 2	PTGS2	75
2	Prostaglandin G/H synthase 1	PTGS1	66
3	Nitric oxide synthase, inducible	NOS2	20
4	Sodium-dependent dopamine transporter	SLC6A3	17
5	5-Hydroxytryptamine receptor 3A	HTR3A	11
6	Peroxisome proliferator activated receptor gamma	PPARG	9
7	Mitogen-activated protein kinase 14	MAPK14	8
8	Transcription factor p65	RELA	7
9	Matrix metalloproteinase-9	MMP9	5
10	72 kDa type IV collagenase	MMP2	4

**Table 4:** Top 7 high-degree active compounds in the network.

No.	Molecule ID	Molecule name	Molecular weight	OB (%)	DL	Herbs	Degree
1	MOL000098	Quercetin	302.25	46.43	0.28	CQZ, CNX, HB,TFL, YHS	19
2	MOL000449	Stigmasterol	412.77	43.83	0.76	CS, HB, TFL, YHS	6
3	MOL012286	Betavulgarin	312.29	68.75	0.39	CNX	6
4	MOL002662	Rutaecarpine	287.34	40.30	0.60	HB	6
5	MOL004328	Naringenin	272.27	59.29	0.21	TFL	6
6	MOL001461	Dihydrochelerythrine	349.41	32.73	0.81	YHS	6
7	MOL001463	Dihydrosanguinarine	333.36	59.31	0.86	YHS	6

OB : oral bioavailability; DL : drug-likeness; CQZ : Cheqianzi; CNX : Chuanniuxi; HB : Huangbo; TFL : Tufuling; YHS : Yanhuosuo; CS : Chishao.

**Table 5:** Molecular docking results of 7 active compounds in TFDC binding with IL-1*β*.

Targets	PDB ID	Molecule ID	Molecule name	Binding energy (kcal/mol)
IL-1*β* (IL1B)	5R8Q	MOL002662	Rutaecarpine	−8.5
		MOL001463	Dihydrosanguinarine	−8.2
		MOL000449	Stigmasterol	−7.6
		MOL004328	Naringenin	−7.4
		MOL000098	Quercetin	−7.2
		MOL001461	Dihydrochelerythrine	−7.0
		MOL012286	Betavulgarin	−6.8

## Data Availability

The data used and analyzed during the current study are available from the corresponding author on reasonable request.
